# Genome-wide association study of post-harvest physiological deterioration in cassava (*Manihot esculenta* Crantz) using visual and AI-powered phenotyping

**DOI:** 10.3389/fpls.2026.1807180

**Published:** 2026-04-22

**Authors:** Kwame Obeng Dankwa, Jorge Luis Luna Melendez, Juan Camilo Giraldo, Camilo Enrique Sánchez-Sarria, Michael Selvaraj, Bunmi Olasanmi, Winnie Gimode

**Affiliations:** 1 Plant Breeding Program, Pan African University, Life and Earth Sciences Institute (Including Health and Agriculture), Ibadan, Oyo State, Nigeria; 2 International Center for Tropical Agriculture (CIAT), Palmira, Colombia; 3 Department of Crop and Horticultural Sciences, Faculty of Agriculture, University of Ibadan, Ibadan, Nigeria

**Keywords:** AI, cassava, genetics, GWAS, PPD, QTL, SNP markers

## Abstract

**Introduction:**

Postharvest physiological deterioration (PPD) is a rapid and severe process in cassava that causes root discoloration and spoilage soon after harvest, limiting shelf life and commercial value of the storage roots. Although environmental interventions can temporarily delay PPD, they are impractical for large-scale use. Previous studies have focused mainly on transcriptome, proteome, and candidate-gene analyses, with a few reporting robust and independently validated quantitative trait loci (QTLs). Identifying PPD-linked-QTLs is essential to breeding PPD-tolerant cassava varieties.

**Methods:**

In this study, a genome-wide association study (GWAS) was conducted to identify genomic regions associated with PPD tolerance using both human visual scoring (VS) and an artificial intelligence (AI)-powered phenotyping method. A mapping population of 298 cassava accessions was genotyped using two platforms: DArTag mid-density panel and genotyping-by-sequencing (GBS).

**Results and discussion:**

Across both genotyping platforms, 6 significant SNPs were identified using VS dataset and 6 using the AI phenotypic dataset. Consistent associations on chromosomes 1 and 12 across both phenotyping and genotyping platforms indicate potentially robust genomic regions influencing PPD response. Overall, the AI-powered phenotyping approach presents a standardized and reproducible PPD scoring procedure over the traditional visual scoring in future breeding programs for PPD. Despite differences in marker density, both DArTag and GBS markers capture comparable insights into the genetic structure of the GWAS panel. These findings provide insights into the genetic basis of PPD in cassava and offer valuable targets for marker-assisted and genomic selection toward developing cassava varieties with delayed PPD.

## 1 Introduction

Cassava (*Manihot esculenta* Crantz) is an important food security crop and a potential industrial crop in the world today. It is cultivated mainly in the tropics for its starch filled storage roots (Lokko et al., 2007). Cassava is a robust crop that can produce relatively reasonable root yield under marginal production conditions such as low soil fertility and irregular rainfall patterns, making it a risk buffer crop for smallholder farmers (Katz and Weaver, 2020; Ramcharan et al., 2017; Burns et al., 2010). It is the second most important staple crop in Sub-Saharan Africa with the highest per capita consumption of about 800g per person/day (Otun et al., 2023). Aside from gaining popularity as a staple food crop around the world, cassava now stands as an industrial and biofuel crop due to the high starch content of its storage roots (Dankwa and Peprah, 2019; Ihemere et al., 2008; Munyikwa et al., 1997). Dried cassava roots can contain more than 80% starch of the total dry weight (Li et al., 2017; Baguma, 2004).

The entire cassava value chain heavily depends on the roots. However, post-harvest physiological deterioration (PPD) is a major constraint to cassava production affecting the root’s marketability and processing quality. The phenomenon is characterized by discoloration of cassava storage roots from the natural white or cream flesh to blue-black or brown usually in response to physical bruise and thereby effectively reducing its quality (Beeching et al., 1998a; Noon and Booth, 1977; Wheatley and Gómez, 1985). This occurrence is swift, initiating a physiologically active response within 15 minutes of root injury (Houmani et al., 2018) and a visible discoloration appearing within 24–72 h of harvest (Iyer et al., 2010; Reilly et al., 2004; Beeching et al., 1998a, 1998b). This sometimes leads to further deterioration from microbial infection, causing roots to rot and soften, thereby rendering them useless (Luna et al., 2021).

Unfortunately, root wounding or mechanical damage is extremely hard to prevent and almost inevitable during cassava harvesting. This problem is exacerbated by the need to transport cassava roots to markets and processing factories which are usually far from the farms, especially in developing countries where road networks are poor and not easily plied (Reilly et al., 2004; Han et al., 2001). At the biochemical level, PPD is mainly caused by reactive oxygen species (ROS) production and accumulation in response to mechanical damage, a phenomenon called oxidative stress (Iyer et al., 2010; Reilly et al., 2004; Huang et al., 2001; Buschmann et al., 2000).

Advances in genomics through next-generation sequencing have led to the development of millions of new markers, which have been consistently utilized in studies of key agronomic traits (Edwards and Batley, 2010). Plant breeders increasingly use modern tools such as marker-assisted selection and genomic selection (GS) to enhance the speed and accuracy of developing crop varieties with desired traits (Crossa et al., 2017). In contrast, phenotype-based recurrent selection remains more challenging in cassava breeding due to its long maturity period (~10–12 months), low multiplication rate, and asynchronous flowering (Ceballos et al., 2012). Genome-wide association studies (GWAS) utilize linkage disequilibrium (LD) and high-density marker sets to dissect the genetic architecture of quantitative traits (Nordborg and Weigel, 2008). It continues to play an important role in plant breeding and has been applied to many phenotypic traits in cassava including carotenoid and dry matter content (Rabbi et al., 2017), disease resistance (Wolfe et al., 2016; Kulakow et al., 2013), starch content, harvest index, leaf shape, and many more morphological and quality traits (Rabbi et al., 2022; Zhang et al., 2018). Nevertheless, there is a limited report on quantitative trait loci for PPD in cassava. Genetic intervention to the PPD problem in cassava is not only crucial but also the best way toward a sustainable cassava production and value chain. Moreover, the labor-intensive and subjective nature of PPD visual scoring presents a challenge, as different evaluators may assign different scores to the same deterioration level under similar conditions. To address this, the CIAT phenomics team developed AI-based algorithms for efficient analysis of large datasets, enabling rapid cassava root screening for PPD symptoms (Ayalde et al., 2024). This approach uses image and color identification techniques and offers a fast, reliable, and standardized method for PPD phenotyping.

Salcedo and Siritunga (2011) suggested that, for an effective solution to PPD in cassava, both basic and applied research should focus on long-term strategies such as developing improved varieties through conventional breeding and selection. However, it will be important to first develop standardized methods for evaluating PPD that are universal in both practice and quantification. A uniform evaluation tool would benefit scientists globally by enabling more accurate correlations between phenotypic PPD tolerance and findings from molecular and cellular studies.

In this study, PPD scoring was conducted using human visual assessment and AI-powered image analysis on 298 cassava genotypes in a clonal evaluation trial over two years at CIAT, Colombia. These clones were also genotyped using two distinct genotyping platforms: DArTag mid density and genotyping by sequencing (GBS). Separate GWAS analyses were performed to identify loci associated with PPD based on these two phenotypic datasets. The findings provide insights into genomic regions linked to PPD in cassava and highlight the potential of AI image analysis for enhanced and uniform PPD phenotyping.

## 2 Materials and methods

### 2.1 Experimental design and field establishment of mapping population

A field evaluation of a multi-parental mapping population comprising 298 genotypes was established in Palmira, Colombia. A partial replication design was used in the field layout. Each genotype was planted in a single-row plot containing six plants, with a spacing of 0.8 meters between plants and 1.2 meters between plots. The field evaluation was carried out in two seasons (2023/2024 and 2024/2025). Harvesting was done 11 months after planting. Although specific plot locations shifted between seasons to accommodate crop rotation, both trials were conducted at the Palmira station. During the 2023/2024 season, the average temperature, relative humidity, and precipitation were 32.75 °C, 80.32%, and 2.43 mm, respectively, compared with 27.07 °C, 80.88%, and 0.07 mm in the 2024/2025 season.

### 2.2 PPD phenotyping

**Figure 1:**
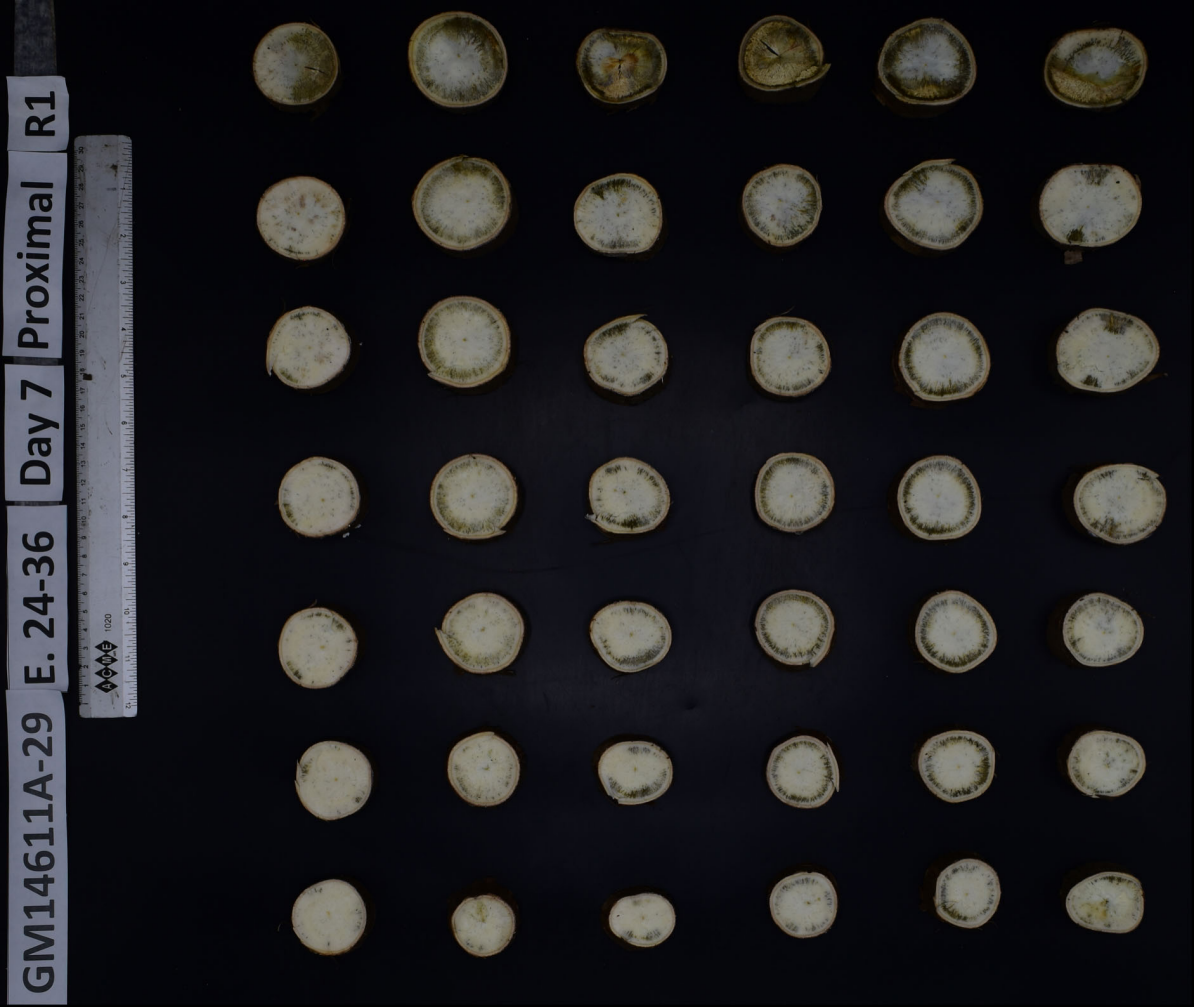
Representative image of cassava root slices for postharvest physiological deterioration (PPD) assessment. Slices are displayed against a high-contrast dark background. These standardized images are used for both human visual scoring and as input data for the AI-powered scoring model to evaluate PPD.

Two approaches were employed to phenotype PPD in this experiment; human visual scoring (VS) and AI-powered image analysis. The VS was carried out according to the method by Wheatley (1982), with some modifications (Dankwa et al., 2025; Sánchez et al., 2013). For each genotype, three plants were harvested and ten commercial-size roots from the three plants were used for PPD phenotyping. To induce deterioration, the distal and proximal extremes of each root were cut to obtain a 15-cm piece. The distal part of the root was covered with a plastic film and secured with a rubber band. The roots were then stored in a controlled chamber and PPD was evaluated on day 7 of storing the prepared roots, starting from the day of harvest as day zero (day 0). The temperature and relative humidity of the chamber were monitored using the Multi-use PDF Temp & RH --TempU03 Data Logger (Luna et al., 2021) and they were kept approximately at 25 °C and 70%, respectively. For the PPD scoring, the stored roots were transversely cut into seven slices (2cm thick), starting at the proximal end. Images for the slices were taken against a dark background (**Figure 1**) using FOTODIOX LED 770 STUDIO BOX and accessories. Each slice was assigned a PPD score between 0 and 10, corresponding to the percentage of the cut surface that exhibits discoloration of the parenchyma (0 = 0%, 1 = 10%, 2 = 20%, etc.) (Luna et al., 2021; Sánchez et al., 2013). The PPD score for each root was calculated using the average of the seven slices. Decomposed or microbial-damaged roots were discarded. The images of the root slices were subjected to a deep-learning high throughput technique using an AI-powered PPD scoring model with the PPDAnalyzer v2.0 (Ayalde et al., 2024).

The AI-powered PPD scoring model (Ayalde et al., 2024) was developed using YOLO (You Only Look Once) foundation models to classify the severity of PPD from digital images. Among the tested architectures, YOLOv8 achieved the highest performance, with a mean average precision (mAP) of 84.0%, demonstrating a good balance between precision (0.77) and recall (0.831). To further improve the accuracy of damage estimation, the workflow incorporated the Segment Anything Model (SAM) for precise background removal and K-means clustering for automated quantification. The model was trained using a dataset of 1,142 images containing 23,934 expert-validated annotations of individual cassava root slices. The dataset was randomly divided into training (80%; 19,152 annotations), validation (15%; 3,584 annotations), and testing (5%; 1,198 annotations) sets to minimize bias and ensure robust model evaluation. Model performance was assessed using confusion matrices, which provided insight into class-specific predictions and misclassifications, and mean absolute error (MAE), which measured the absolute difference between the AI-predicted PPD percentage and the expert-assigned ground truth (Ayalde et al., 2024).

#### 2.2.1 Phenotypic data analysis

For each PPD dataset (VS and AI), two years data were collapsed into a single best linear unbiased prediction (BLUPs) using a linear mixed model Yijk=μ+βj +gi +(gβ)ij +rk(j) +ϵijk within the lme4 package in RStudio (Boer and Rossum, 2022; R Core Team, 2021). Where: Yijk = observed PPD value for the i-th genotype, in the j-th year, and k-th rep. μ = overall intercept (grand mean). βj = fixed effect of the j-th year (Year is fixed). gi = random effect of the i-th genotype. (gβ)ij = genotype x environment (year) interaction. rk(j) = random effect of the k-th replication nested within the j-th year. ϵijk = residual error.

The BLUPs were used to calculate the PPD genetic correlation using three models (MLMM, BLINK, and FarmCPU) in GAPIT (Wang et al., 2022). The same lme4 package was used to estimate PPD broad sense heritability as: H^2^ $=\frac{\sigma_{G}^{2}\;}{\sigma_{G}^{2}\;+(\sigma_{GE}^{2}/e)+(\sigma_{E}^{2}/re)}$ Where: H^2^ is the broad-sense heritability, $\sigma_{G}^{2}$ is genotypic variance, $\sigma_{GE}^{2}$ is genotype × environment (year) interaction variance, $\sigma_{E}^{2}$ is Residual (error) variance, *e* is number of environments (years), *r* is number of replications per environment (year).

To calculate the coefficient of variation (CV) for each year, a mixed linear model $Y_{ik}=\mu +G_{i}+R_{k}+\epsilon_{ik}$ was fitted, where $Y_{ik}$ is the observed PPD value of the *i*-th genotype in the *k*-th replication, *μ* is the overall mean, $G_{i}$ is the random genotypic effect, $R_{k}$is the random replication effect, and $\epsilon_{ik}$ is the residual error. The CV for each year was calculated as the ratio of the residual standard deviation to the year-specific trait mean.

Pearson’s correlation coefficient (*r*) was used to assess the relationship between human visual and AI-based PPD scores. Statistical significance was tested at *P* < 0.05. The result was visualized using ggplot in R.

### 2.3 DNA extraction and genotyping

Genomic DNA was extracted from cassava leaf samples using the CTAB method, following the DNA extraction protocol by Doyle (1991) with some modifications. DNA quantification was performed via absorbance reading using a Nanodrop spectrophotometer. All DNA samples were assessed on a 1% agarose gel to verify quality before sequencing. The mapping population was genotyped using two distinct genotyping platforms; DArTag mid density panel and genotyping by sequencing (GBS) to identify single nucleotide polymorphism (SNP) markers associated with PPD in cassava. The DArTag mid density genotyping was done at Intertek, Australia while the genotyping by sequencing (GBS) was done at University of Minnesota Genomics Center.

### 2.4 SNP calling and filtering

For DArTag mid density, which is based on the *Manihot esculenta* version 7.1 genome, 3,002 single nucleotide polymorphisms (SNP) markers were obtained. These SNPs were filtered to exclude those with missing data ≥80% and a minor allele frequency (MAF) < 0.05, using the snpReady package in RStudio (Granato et al., 2018; R Core Team, 2021). For genotyping-by-sequencing (GBS), the raw paired-end sequence data trimming and resynchronization were performed on each sample using the gbstrim and resync Perl scripts. All sample sequences were then aligned and indexed to version 8.1 of the *Manihot esculenta* reference genome using bwa mem (version 0.7.12), and variant discovery was carried out following the GATK Best Practices workflow (version 4.1.8) (DePristo et al., 2011), retaining only biallelic SNPs. The resulting dataset was filtered with vcftools (Danecek et al., 2011) using thresholds of 10% missing data, a minor allele frequency (MAF) of at least 5%, and a quality score (Q) greater than 30. After these filtering processes, 2,526 SNPs for the DarTag dataset and 30,046 SNPs for the GBS dataset were utilized to conduct a GWAS.

### 2.5 Linkage disequilibrium, population structure, and genetic relatedness analysis

To assess genome-wide marker distribution, SNP density was profiled for both DArTag and GBS datasets using a 1-Mb sliding window with the SRplot platform (Tang et al., 2023). For each genetic dataset (DArTag and GBS), linkage disequilibrium decay (LD decay) was calculated using PLINK version 1.9 (Purcell et al., 2007) and visualized with the ggplot2 R package (R Core Team, 2021). To account for genetic relationships between individuals, kinship was calculated with the ZHANG (Zones Harbored Adjustments of Negligent Genetic) algorithm in GAPIT (Zhang, 2014; Wang et al., 2022). To reveal potential subgroups within the population, principal component analysis (PCA) was performed using the genotypic marker datasets in TASSEL software (version 5.2.96) and visualized using the ggplot2 R package. Genotypes were categorized by common maternal parental background to provide biological context to the observed genetic clustering.

### 2.6 Association analyses and candidate gene exploration

The phenotypic (BLUP values) and genotypic data were analyzed to determine associations using the Genomic Association and Prediction Integrated Tool (GAPIT) package version 3.4.0 in R employing three models (MLMM, BLINK, and FarmCPU) (Wang and Zhang, 2021). Significant marker–trait associations were identified using a Bonferroni-corrected significance threshold (*P* < 0.05/*m*), where *m* represents the total number of SNPs tested. The identified significant loci were explored for potential candidate genes using the *Manihot esculenta* v7.1 and v8.1 for DArTag mid-density and GBS, respectively. The corresponding v8.1 positions for DArTag v7.1 positions were explored as well.

The effectiveness of the models in controlling for systematic bias was evaluated by calculating the genomic inflation factor(λ). Defined as the ratio of the median of the observed χ2 statistics to the expected median χ2 under the null hypothesis. The P-values generated by GAPIT for each model were imported into RStudio to calculate the chi-square statistics and the resulting λ values.

## 3 Results

### 3.1 Phenotypic distribution

**Figure 2:**
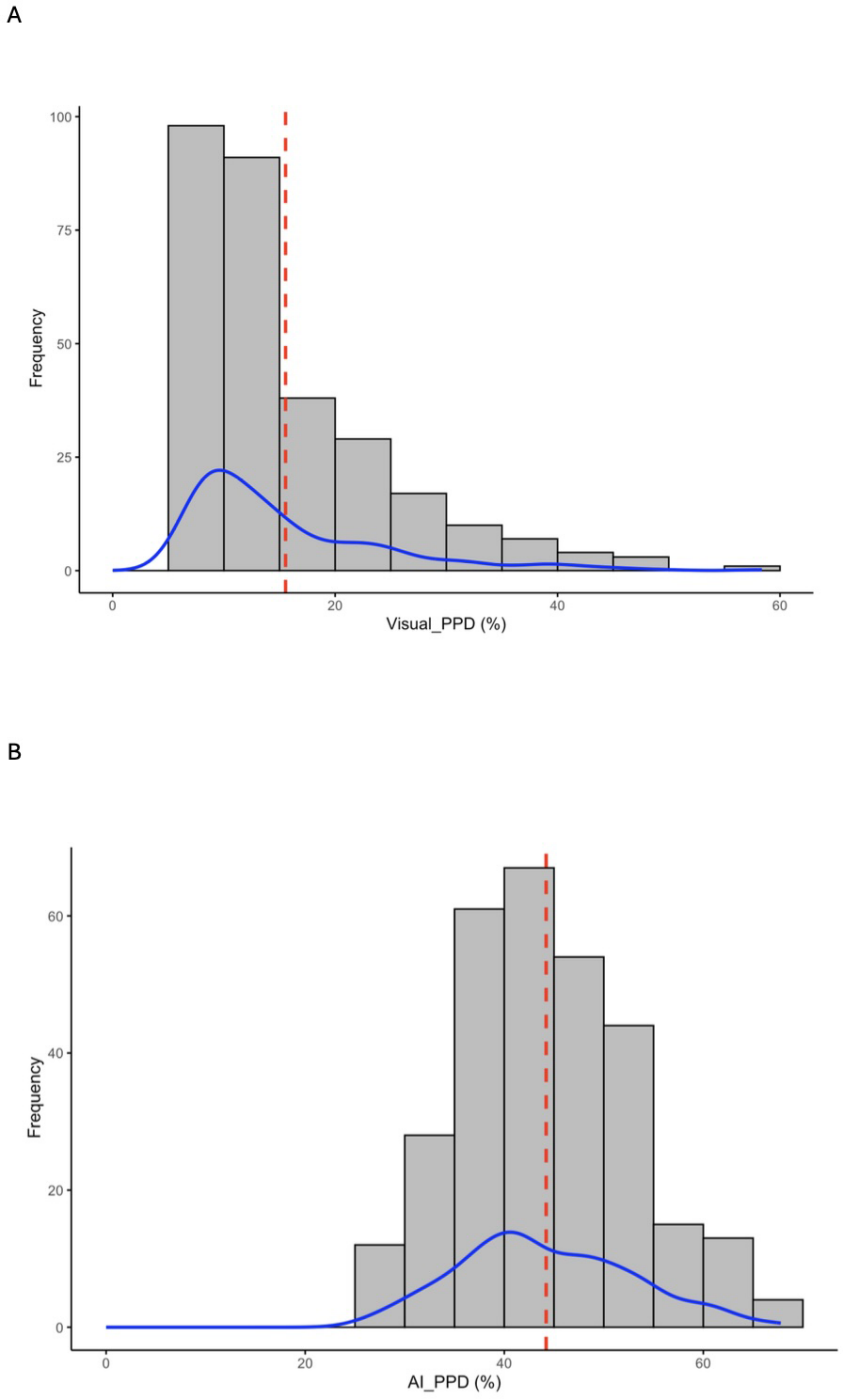
Distribution of PPD severity via human visual and AI-powered phenotyping. Histograms with density curves (blue) and mean lines (dashed red) showing the distribution of PPD severity percentages across the population. **(A)** Human visual scoring shows a distribution skewed toward lower severity levels. **(B)** AI-powered scoring based on image analysis shows a broader distribution with a higher mean severity.

A comparison of the phenotypic data distributions for PPD scores obtained from human visual assessment (**Figure 2A**) and AI-powered image analysis (**Figure 2B**) revealed similarities and important methodological differences. The distribution of visually scored PPD was right skewed, with most genotypes clustering toward the lower end of the severity scale. The majority of observations fell between 0 – 20%. The mean visual PPD score was 15.47% and the distribution exhibited a long tail extending toward higher values (>50%).

In contrast, the AI-powered phenotyping method produced a more symmetrical, near-normal distribution centered around moderate PPD levels. The AI-based PPD values ranged approximately from 20 to 75%, with most genotypes clustering near the mean of 43.84%. Unlike the visual score distribution, the AI-based scores occupied a broader range. The mean line fell near the center of the distribution, confirming a more even spread of phenotypes across the panel.

Across both approaches, the visual scoring method assigned lower percentage values for PPD severity relative to the AI-based system, particularly for genotypes exhibiting mild-to-moderate deterioration.

For visual PPD scores, the calculated broad-sense heritability was high (H^2^ = 0.71), despite high coefficients of variation (55.5% in 2023/24 and 73.2% in 2024/25). In contrast, AI-based scores showed lower H^2^ (0.64) but significantly more stable CV values (35.2% and 45.7%). Visual and AI-based PPD BLUPs were positively correlated (*r* = 0.55; **Supplementary Figure 1**). The elevated CV values likely reflect the inherent variability of PPD, which often manifests as significant variation between replicates of the same genotype or even among individual roots harvested from the same plant.

### 3.2 Genomic structure and landscape in the studied population

#### 3.2.1 SNP density across the cassava genome

**Figure 3:**
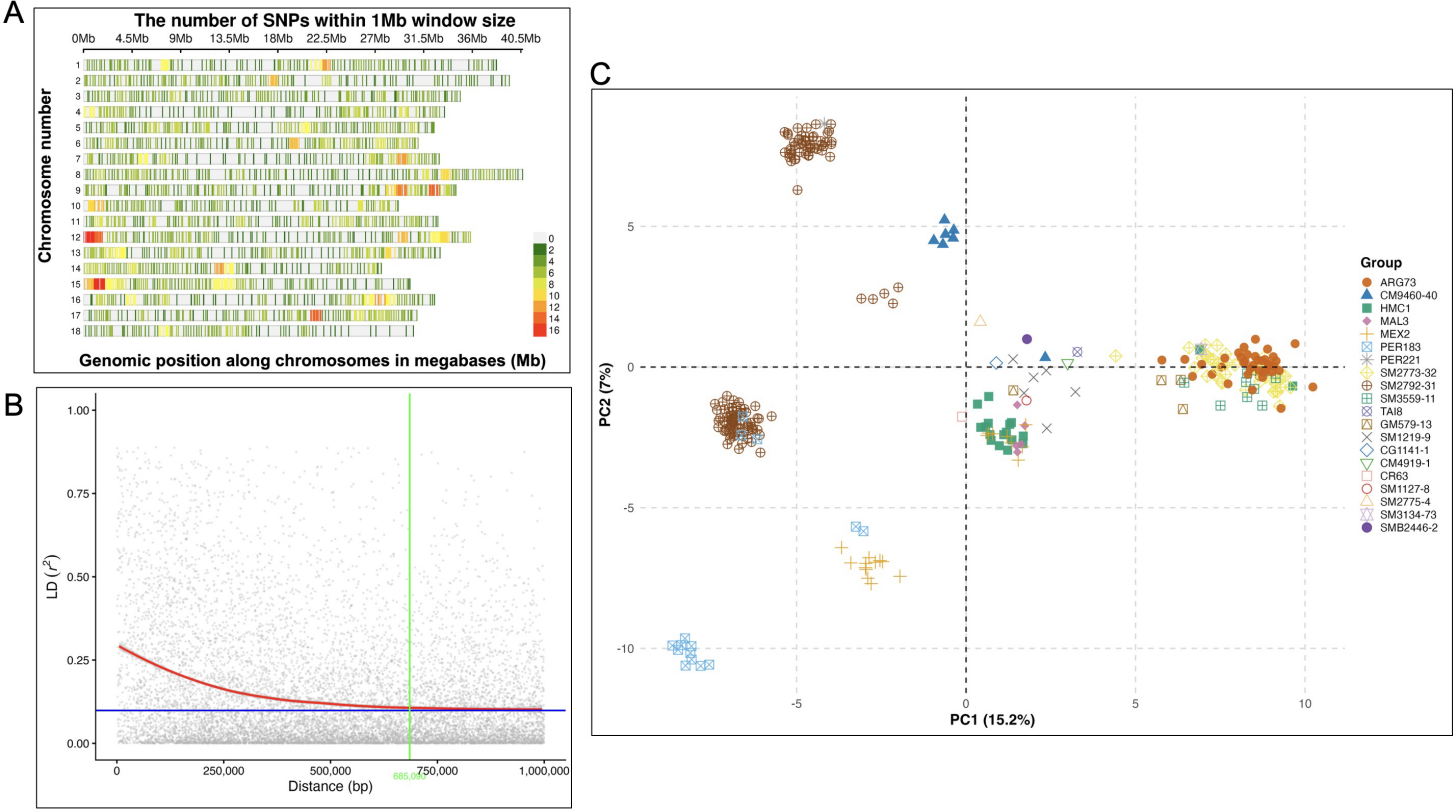
Population structure of 298 cassava genotypes used in genome-wide association studies (GWAS) based on DArTag markers. **(A)** Marker distribution and density across the 18 chromosomes, **(B)** linkage disequilibrium decay, and **(C)** clustering results visualized on a principal component analysis (PCA) plot.

**Figure 4:**
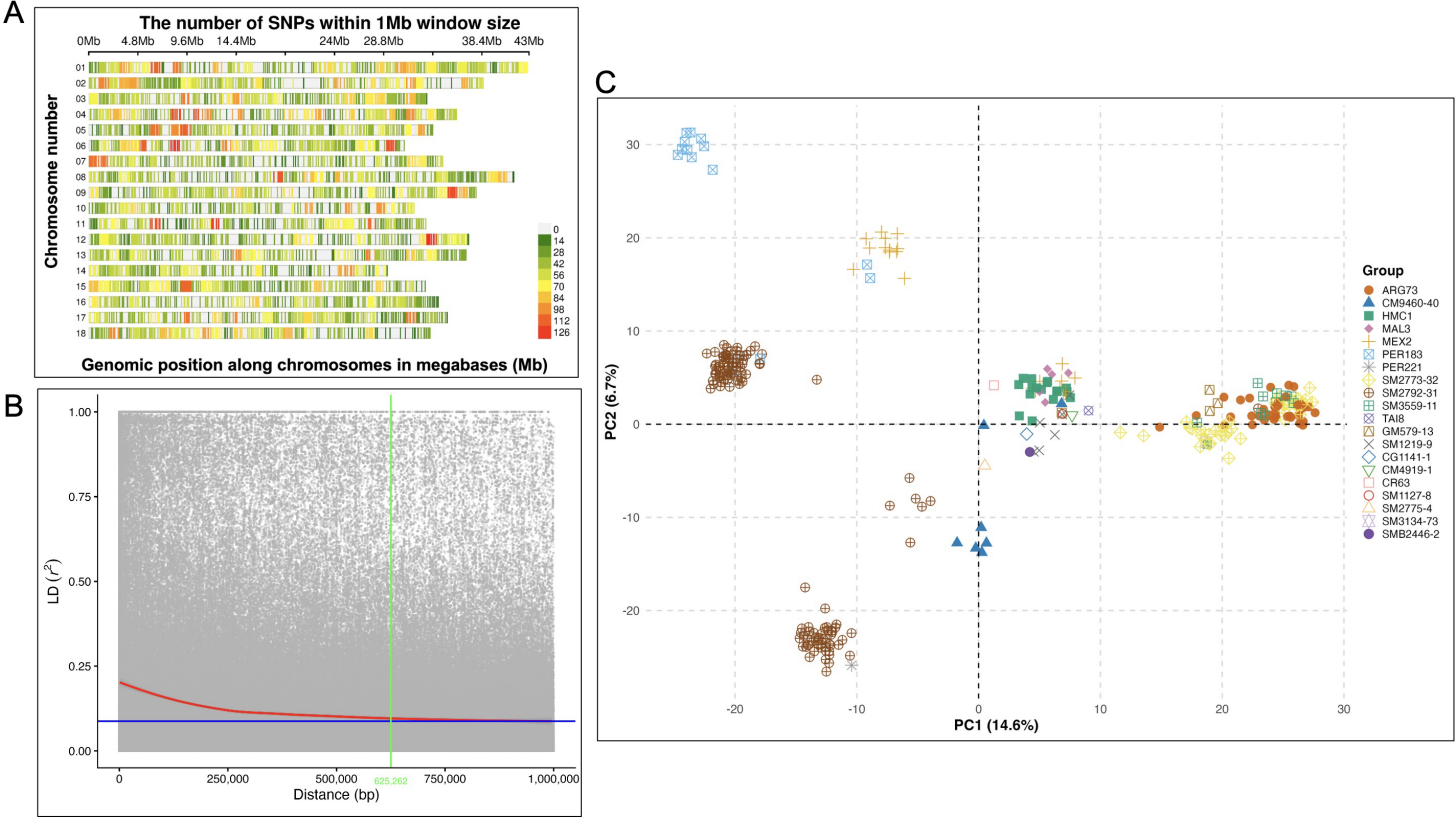
Population structure of 298 cassava genotypes used in genome-wide association studies (GWAS) based on GBS markers. **(A)** marker distribution and density across the 18 chromosomes, **(B)** linkage disequilibrium decay, and **(C)** clustering results visualized on a principal component analysis (PCA) plot.

To assess genome-wide marker distribution, SNP density was profiled for both DArTag and GBS datasets using a 1-Mb sliding window (**Figures 3A**, **4A**). The DArTag markers (**Figure 3A**) exhibited a moderate and localized genomic distribution, with SNP density peaking at 16 SNPs/Mb. While higher densities were noted on some chromosomes such as chromosomes 12 and 15, large segments of the genome remained sparsely covered. This distribution reflects the targeted nature of the DArTag panel, where marker density is a function of probe design and the prioritization of specific loci. In contrast, GBS markers (**Figure 4A**) provided a significantly higher marker volume, with a nearly 8-fold increase in peak density (up to 126 SNPs/Mb). However, despite the higher density, GBS maintained a non-uniform distribution, with prominent marker-dense regions on chromosomes 6, 8, 11, and 14, where windows exceeded 98 SNPs/Mb, with some regions showing reduced marker density. The GBS distribution reflects the inherent bias of restriction-site-associated DNA sequencing, where marker density depends on the frequency and accessibility of restriction enzyme recognition sites. Overall, GBS provided greater genomic resolution which was approximately 6 to 8-fold higher SNP counts per window compared to DArTag – while DArTag provided targeted coverage of high-priority loci at a lower density.

#### 3.2.2 Linkage disequilibrium decay patterns

Linkage disequilibrium (LD) decay analysis revealed highly consistent patterns across both the DArTag and GBS marker platforms (**Figures 3A**, **4B**). The smoothed r² curve intersected the background LD threshold (r² ≈ 0.10) at approximately 685,090 bp for DArTag and 625,262 bp for GBS, suggesting that both platforms are effectively capturing the same underlying genomic architecture and recombination landscape of the population. The scatter patterns and fitted curves indicate that both datasets capture strong LD at short distances, followed by a gradual and comparable decline over larger genomic distances. While GBS exhibited a marginally steeper initial decline, offering a slightly refined resolution for identifying narrow QTLs, the overall widespread recombination indicated in both panels suggests that both datasets are well-suited for robust association mapping in this cassava germplasm.

#### 3.2.3 Principal component analysis

Principal component analysis (PCA) was used to assess genetic structure in the cassava panel using DArTag (**Figure 3C**) and GBS markers (**Figure 4C**). The analysis revealed a high level of congruency in clustering patterns between the DArTag and GBS marker platforms. In both analyses, the first two principal components accounted for a comparable proportion of the total genetic variance (21.3-22.2%), indicating similar large-scale population structure across marker systems. Both platforms resolved the same primary genetic groupings with accessions generally clustered according to shared parental background. Notably, groups such as ARG73, PER183, CM9460-40, HMC1, SM3559-11, and SM2773–32 formed well-defined clusters across both marker systems, though some overlap was also observed among certain families. While the absolute orientation of the clusters shifted along the axes—a common artifact of PCA coordinate calculation—the relative genetic distances between the families remained stable.

In the DArTag analysis, PC1 and PC2 explained 15.2% and 7.0% of the variance, respectively, while the GBS-based PCA explained 14.6% of the variance in PC1 and 6.7% in PC2, closely mirroring the DArTag results. Despite slight differences in resolution, the overall clustering remained comparable between platforms, confirming that both DArTag and GBS are robust for population structure characterization.

Notably, the SM2792–31 maternal group appeared as two distinct subgroups. This separation is attributed to the use of different paternal parents (PER183 and PER221) in crosses with the SM2792–31 female parent. The PCA effectively captured this paternal influence, splitting the maternal family into two discrete clusters positioned toward their respective paternal donors. This further confirms that both DArTag and GBS were robust and sensitive enough to resolve pedigree-level substructure.

### 3.3 Marker loci linked to PPD

**Figure 5:**
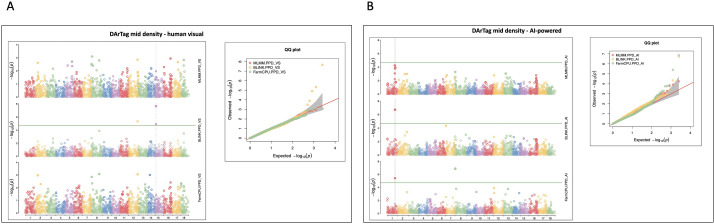
Manhattan and quantile–quantile (Q-Q) plots of significant markers for PPD from the GWAS analysis conducted on 298 cassava accessions and 2,526 DArTag mid density SNP markers; **(A)** human visual PPD score, **(B)** AI-powered PPD scores.

The GWAS conducted using both DArTag mid-density and GBS markers identified loci associated with post-harvest physiological deterioration (PPD) under human visual and AI-powered phenotyping approaches. The DArTag-based GWAS revealed several significant SNPs distributed across multiple chromosomes for both scoring methods (**Table 1**; **Figure 5**). For the human visual dataset, significant associations were detected on chromosomes 12 and 15 with the SNPs CassV7_chr12_32503122 and CassV7_chr15_3648525 explaining 5.18% and 0.75% of the phenotypic variance, respectively. The AI-powered scoring approach identified loci on chromosomes 1 and 8, with CassV7_chr08_901823 explaining 0.96% of the phenotypic variance, and the other associated locus on chromosome 1 exhibiting negligible (0%) individual PVE values (**Table 1**). The Manhattan plots (**Figures 5A, B**) displayed clear peaks corresponding to these associations, while the Q-Q plots indicated proper control of population structure. However, the BLINK model exhibited mild genomic inflation (λ = 1.22) for the AI-powered assessment, though the SNP (CassV7_chr01_28520241) was corroborated by the more conservative FarmCPU model (λ = 0.92).

**Table 1:** Significant SNPs identified from human visual and AI-powered PPD scoring methods using DArTag mid density markers with genomic inflation factor (λ) for each model.

PPD scoring method	Model	SNP_ID	Chromosome	Position (v7.1)	Position (v8.1)	P. value	Minor allele frequency	Phenotypic variance explained (%)
Visual	BLINK (λ = 0.97)	CassV7_chr12_32503122	12	32503122	33211138	4.60E-06	0.32	5.18
		CassV7_chr15_3536121	15	3536121	3393477	2.24E-08	0.47	0
		CassV7_chr15_3648525	15	3648525	3505542	1.24E-05	0.36	0.75
AI-powered	BLINK (λ = 1.22)	CassV7_chr01_28520241	1	28520241	32684118	1.92E-07	0.18	0
	FarmCPU (λ = 0.92)	CassV7_chr01_28520241	1	28520241	32684118	3.97E-06	0.18	0
		CassV7_chr08_901823	8	901823	647907	1.37E-07	0.35	0.96

**Figure 6:**
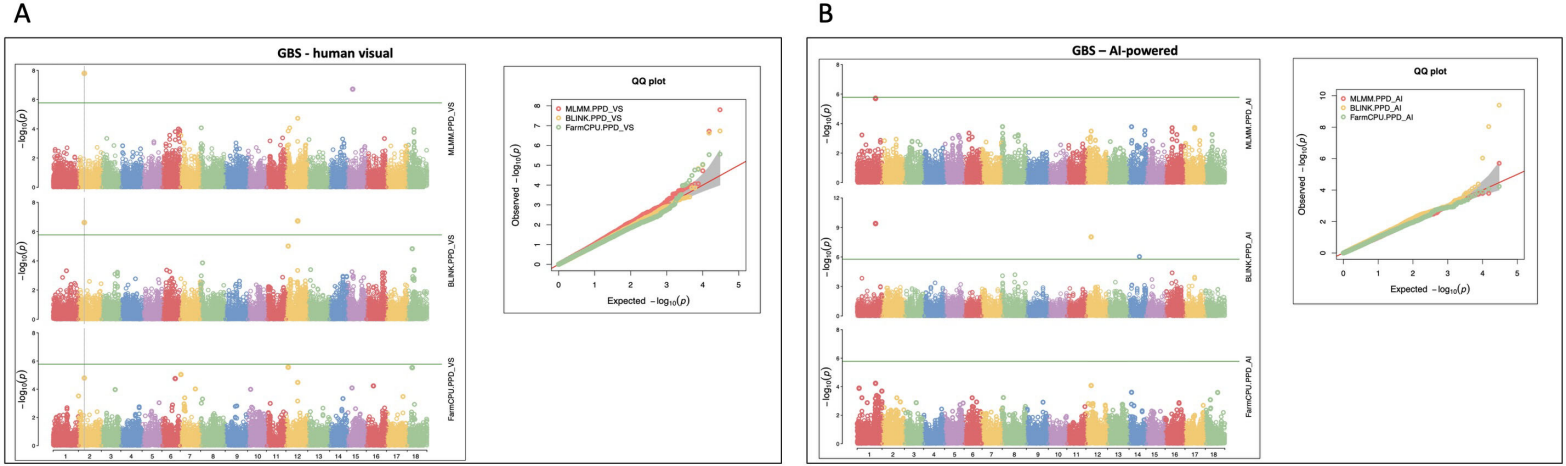
Manhattan and quantile–quantile (Q-Q) plots of significant markers for PPD from the GWAS analysis conducted on 298 cassava accessions and 30,046 genotyping by sequencing (GBS) SNP markers; **(A)** human visual PPD scores, **(B)** AI-powered PPD scores.

Similarly, GWAS using GBS markers identified several significant SNPs associated with PPD across the two phenotyping methods (**Table 2**; **Figure 6**). For the human visual assessment, major association peaks were observed on chromosomes 2, 12 and 15 with S02_9881815 consistently detected across MLMM and BLINK models. This marker explained a high phenotypic variance of 35.5% and 44.8% for MLMM and BLINK models respectively. The AI-powered dataset revealed additional significant loci on chromosomes 1, 12, and 14 with S14_17899979 explaining phenotypic variance of 57.7%. The Manhattan and Q-Q plots (**Figures 6A, B**) confirmed these associations and demonstrated good model fit with limited false positives. Notably, the BLINK model for AI-powered traits showed mild genomic inflation (λ = 1.18) while the visual scoring model exhibited a more conservative, slightly deflated value (λ = 0.87).

**Table 2:** Significant SNPs identified from human visual and AI-powered PPD scoring methods using GBS markers with genomic inflation factor (λ) for each model.

PPD scoring method	Model	SNP_ID	Chromosome	Position (v8.1)	P. value	Minor allele frequency	Phenotypic variance explained (%)
Visual	MLMM (λ = 1.01)	S02_9881815	2	9881815	1.59E-08	0.09	35.95
		S15_9885471	15	9885471	1.92E-07	0.11	17.41
	BLINK (λ = 0.87)	S02_9881815	2	9881815	2.38E-07	0.09	44.80
		S12_20118969	12	20118969	1.86E-07	0.08	9.45
AI-powered	BLINK (λ = 1.18)	S01_31747016	1	31747016	4.02E-10	0.06	21.30
		S12_8904674	12	8904674	9.21E-09	0.48	6.75
		S14_17899979	14	17899979	9.26E-07	0.108	57.70

## 4 Discussion

Artificial intelligence (AI) has had an exciting application in modern plant breeding, both demonstration and potential; data mining, phenotyping, monitoring, multi-omics, environment, sustainability, and economics (Farooq et al., 2024; Sangjan et al., 2025). In winter wheat breeding, artificial intelligence (AI) serves as a link between scientific innovation and practical application by addressing limitations of traditional statistical approaches and enabling the generation of high-quality phenotypic data (Chang-Brahim et al., 2024). Successful genome-wide association studies (GWAS) rely on accurate and reliable phenotypes, which AI facilitates through high-throughput remote phenotyping platforms (Huang et al., 2025). Beyond phenotyping, AI and machine learning have also emerged as essential tools for integrating heterogeneous data sources, including single-cell omics and protein–interaction networks, to identify disease drivers (Hazelett, 2025). The strategy of utilizing AI-derived phenotypes for GWAS is established in recent literature across diverse crops. For example, deep learning models were successfully employed in lettuce to map 11 QTLs linked to seven distinct seed traits (Seki and Toda, 2022). This study demonstrated the effectiveness of image-based phenotyping in accelerating the discovery of the genetic basis for small, complex morphological features—such as seed size and shape—that are difficult to quantify manually. Similarly, research in soybean has utilized deep learning to capture complex phenotypic traits directly from field imagery. Rairdin et al. (2022) compared image-based phenotypic data with manually collected visual scores for GWAS and obtained highly comparable results which were validated by detecting genomic regions near previously reported loci associated with sudden death syndrome in soybean.

The method used to evaluate a trait is critical, as it can influence subsequent decisions made on that trait. Variability in phenotyping methods, particularly differences between specific-gravity and oven-drying approaches for estimating dry matter content (DMC) has been shown to reduce the predictive accuracy of DMC in genomic selection studies (Esuma et al., 2021). The two phenotyping methodologies employed in this study clearly demonstrated the effect of methodology. The human visual PPD scores seem to indicate that visible deterioration symptoms were generally mild during the assessment period and a right-skewed distribution suggests the presence of a smaller subset of highly susceptible genotypes (**Figure 2A**). With the AI-based PPD scores, a broader spread and nearly even distribution with a higher mean indicates higher incidence of PPD. This pattern implies that the AI system may be more sensitive to subtle deterioration features and therefore produces greater phenotypic differentiation among genotypes.

The visual PPD scoring is labor-intensive and more prone to subjectivity or human errors that may further limit the power to detect genomic regions underlying the trait—a challenge that AI-based image analysis is designed to overcome. These contrasting distribution patterns indicate that although both methods capture meaningful biological variation, the AI-based scoring may provide enhanced phenotypic resolution, which is likely beneficial for downstream GWAS, genomic selection, or association mapping analyses. The complexity of human visual scoring likely contributes to the fact that limited QTL for PPD in cassava have been reported (Cortés et al., 2002), with subsequent research relying largely on transcriptomic, proteomic, and candidate-gene evidence rather than robust, replicated QTLs.

The positive association observed between visual PPD BLUPs and AI-derived PPD BLUPs (Pearson’s *r* = 0.55) (**Supplementary Figure 1**) indicates a general agreement between the two approaches, with higher visual PPD scores corresponding to higher AI-predicted PPD values. Despite this positive relationship, some dispersion around the fitted regression line was evident, suggesting that the two methods are not perfectly concordant. The marked experimental variability observed between seasons underscores the significant environmental dependence of PPD expression. Consequently, multilocation and multiseasonal evaluation are essential to ensure the robustness and stability of PPD phenotyping data.

PPD is one of the most important constraints in cassava production (Naziri et al., 2014; Sayre et al., 2011). It is uniquely rapid in cassava, commencing a few hours after harvesting, especially when the storage roots are injured during harvesting and post-harvest handling. The early phase of PPD, usually referred to as the primary stage, is basically a complex physiological and biochemical response that is triggered by root injury and begins at the wound site a few hours after injury (Bayoumi et al., 2008; Reilly et al., 2004). Root wounding or mechanical damage is extremely hard to prevent and almost inevitable during cassava harvesting and transportation. Hence, identifying QTLs linked to delay in PPD and deploying them through marker-assisted selection (MAS) could strengthen the cassava-value-chain through rapid breeding pipelines to reduce postharvest losses.

Markers from the two genotyping platforms used in this study yielded congruent and comparable results. Differences in SNP density reflect the inherent contrasts between DArTag and GBS sequencing approaches (**Figures 3A**, **4A**) and have implications for downstream analyses such as LD decay, haplotype detection, and GWAS resolution. However, despite these differences in marker density and distribution, both platforms revealed similar LD decay patterns. Linkage disequilibrium in both datasets declined with physical distance, reaching a stable r^2^ plateau (0.10) at approximately 625–685 kb. This indicates similar haplotype block structure and recombination rates across both marker platforms (**Figures 3B**, **4B**). This harmony suggests that both DArTag and GBS provide consistent estimates of genome-wide LD structure, supporting their joint use and comparability in downstream analyses, including GWAS and population structure inference.

The DArTag and GBS marker analyses identified unique genomic regions associated with PPD tolerance. The identified loci using AI-based PPD scores highlights the possibility of using AI-assisted phenotyping approaches in capturing PPD-related genetic variation in cassava. While the GBS significant SNPs with high PVE (S01_31747016, S02_9881815, S14_17899979: **Table 2**) met our minor allele threshold (MAF ≥ 0.05), they exhibit limited allele representation in our population. Specifically, the alternate or favorable alleles were primarily present in the heterozygous state and were rarely found in the homozygous state. This likely contributed to inflated PVE estimates and the observed genomic inflation observed especially in the BLINK model (λ = 1.18 – 1.22). While these signals clearly identify PPD-associated regions, further validation in larger, independent populations is required to confirm the magnitude of these genetic effects.

As a biochemical response, PPD is linked to a build-up of reactive oxygen species (ROS) resulting in oxidative stress (Reilly et al., 2004) and enzymes such as catalase, peroxidase, and superoxide dismutase that regulates the ROS levels (Buschmann et al., 2000; Iyer et al., 2010; Reilly et al., 2001). In plants, ROS are produced as a by-product of aerobic respiration and play crucial roles in essential processes such as photosynthesis and other cellular metabolism (Singh et al., 2016; Apel and Hirt, 2004). Under typical conditions plants possess several mechanisms to neutralize ROS, preventing or mitigating their toxicity (Choudhary et al., 2017). However, when under stress (biotic or abiotic), the rate of ROS production far supersedes the rate at which they are dissipated (also called detoxification), causing a disequilibrium and a rapid buildup of ROS, a phenomenon known as oxidative burst (Apostol et al., 1989). This disequilibrium between ROS production rate and detoxification can result in DNA, proteins, and lipids damage (Tripathy and Oelmüller, 2012). In well studied grain crops like wheat and maize, biotic and abiotic factors that could result in large production of ROS include but not limited to drought, extreme temperatures, salinity, ultraviolet‐B radiation, heavy metals, and pathogens (Tripathi et al., 2017; Singh et al., 2016; Arora et al., 2002). In cassava, cultivars with high levels of substances that reduce ROS accumulation such as *β*- carotene (Sanchez et al., 2005) show delayed PPD while overexpression of a cyanide-resistant terminal oxidase in plants (alternative oxidate) that reduces cyanide accumulation has similar effect (Zidenga et al., 2012).

While the candidate genes identified are biologically plausible based on their co-mapping with significant SNPs (**Table 3**), they remain putative until functional validation can be performed. Generally, these annotations (**Table 3**) point to a model in which wound-triggered signaling (Ca²^+^ fluxes and ion channels), vacuolar trafficking and proteostasis, and RNA surveillance contribute to the early cellular responses that may influence PPD occurrence and progress. Cassava PPD is characterized by rapid production and accumulation of reactive oxygen species (ROS), calcium (Ca²^+^) signaling, and programmed cell death (PCD). Ca²^+^-related genes are noteworthy candidates, as cyclic-nucleotide-gated channels potentially mediate Ca²^+^ influx that can regulate ROS generation and PCD soon after harvest, linking external stress cues to internal deterioration processes (Wang et al., 2021; Djabou et al., 2017).

**Table 3:** Putative gene annotations of significant SNPs directly mapped to annotated genes using DArTag and GBS markers, aligned to cassava reference genome versions 7.1 and 8.1, respectively.

PPD scoring type	SNP platform	SNP ID	Chromosome	Position	Gene ID	Gene annotation
Visual	DArTag	CassV7_chr12_32503122	12	32503122 (v7.1)	Manes.12G126100	KINASE INTERACTING (KIP1-LIKE) FAMILY PROTEIN
		CassV7_chr15_3536121	15	3536121 (v7.1)	Manes.15G043900	F19P19.6 PROTEIN
		CassV7_chr15_3648525	15	3648525 (v7.1)	Manes.15G046000	OS02G0131700 PROTEIN
AI-powered		CassV7_chr01_28520241	1	28520241 (v7.1)	Manes.01G133800	DUF1195 family protein
AI-powered	GBS	S01_31747016	1	31747016 (v8.1)	Manes.01G121600	Nonsense-mediated mRNA decay protein 3 (NMD3)

Based on Arabidopsis functional annotation, the F19P19 protein is putatively associated with plant defense responses through transcriptional regulation of defense-related genes and is hypothesized to play a role in innate immune signaling pathways (Reiser et al., 2024). The OS02G0131700 protein belongs to the DUF794 domain family, which is predominantly found in plants and remains functionally uncharacterized (Finn et al., 2016). Some annotation databases further associate this protein with the RNA recognition motif (RRM) family, particularly the RRM1 subtype. RRM-containing proteins in rice have been widely implicated in post-transcriptional regulation and responses to environmental stresses, including pathogen infection, suggesting a potential role for this gene in stress-related regulatory processes (Zhang et al., 2016; Lorković, 2009).

The link with an NMD3-like gene points toward RNA quality control as a potential factor influencing PPD. The NMD genes help regulate faulty or stress-related RNA molecules, and changes in their activity can alter how plants respond to stress (Nyikó et al., 2013). Hence changes in NMD activities can reprogram defense and stress pathways and could therefore influence the magnitude or timing of PPD responses.

Ultimately, the SNPs and candidate genes associated with these diverse biological pathways underscore the complex polygenic nature of cassava PPD. While these specific mechanisms require further functional validation, their identification through congruent GWAS signals may provide a robust genetic framework for future breeding efforts.

## 5 Conclusion

This study provides valuable insights into the genetic basis of post-harvest physiological deterioration (PPD) in cassava using two different phenotypic and genotypic approaches. The AI-based approach may serve as a useful, objective tool for standardizing PPD evaluation in high-throughput breeding contexts. The loci and candidate genes identified provide a plausible framework for understanding PPD tolerance. Collectively, these findings contribute to the genomic resources available for developing cassava varieties with improved post-harvest stability.

## Data Availability

The datasets presented in this study can be found in online repositories. The names of the repository/repositories and accession number(s) can be found in the article/**Supplementary Material**.
