# Supplementary figures and images for: Genome-wide association study of post-harvest physiological deterioration in cassava (Manihot esculenta Crantz) using visual and AI-powered phenotyping
Source: Front Plant Sci. 2026 Apr 22;17:1807180. doi: 10.3389/fpls.2026.1807180 (PMC13144131; doi:10.3389/fpls.2026.1807180)

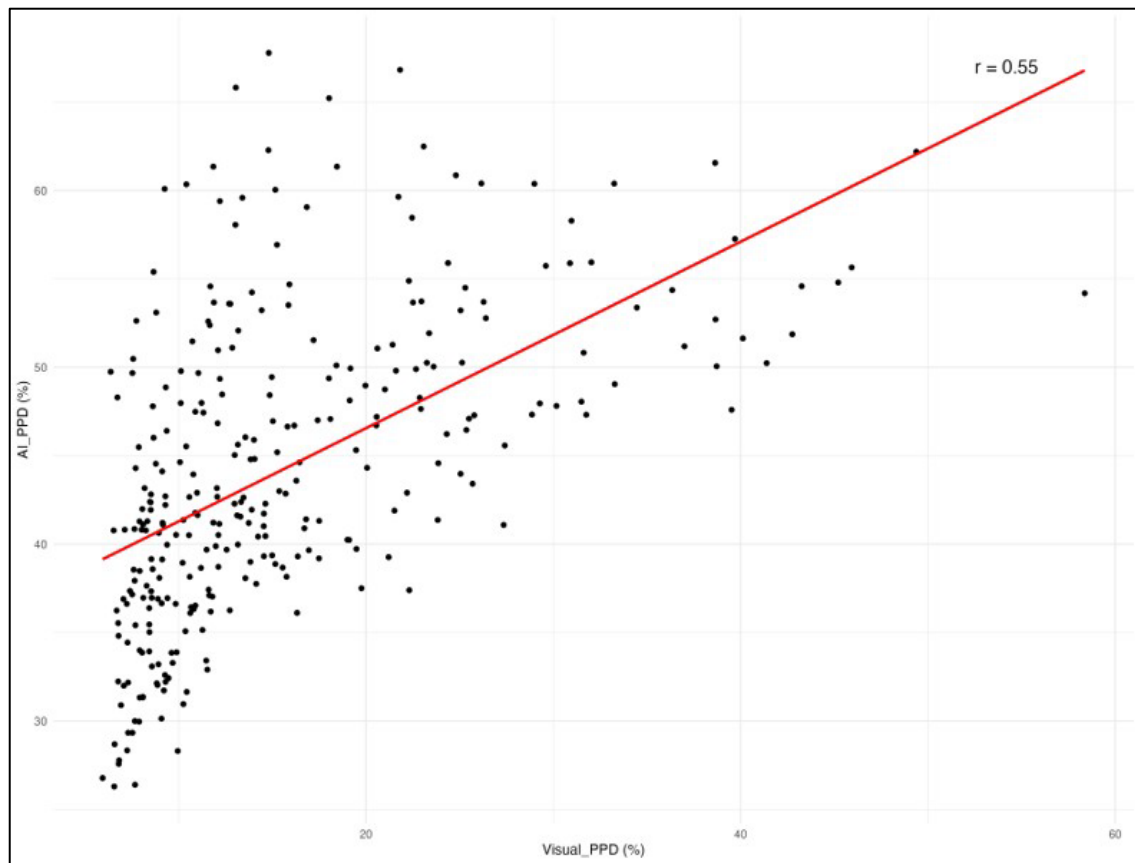

Supplementary Figure S1. Correlation between AI-powered and human visual PPD scores

Supplement: Supplementary file 1 [file DataSheet1.pdf]
